# Supplementary material for: Fetal growth delay caused by loss of non-canonical imprinting is resolved late in pregnancy and culminates in offspring overgrowth
Source: eLife. 2024 May 30;13:e81875. doi: 10.7554/eLife.81875 (PMC11139480; doi:10.7554/eLife.81875)
Supplement: MDAR checklist [file elife-81875-mdarchecklist1.pdf]

eLife
Westbrook Centre
Milton Road
Cambridge CB4 1YG, UK
P 01223 855340
W elifesciences.org
T @elife

Materials Design Analysis Reporting (MDAR) Checklist for Authors

The MDAR framework establishes a minimum set of requirements in transparent reporting mainly applicable to studies in the life sciences.

eLife asks authors to **provide detailed information within their article** to facilitate the interpretation and replication of their work. Authors can also upload supporting materials to comply with relevant reporting guidelines for health-related research (see EQUATOR Network), life science research (see the BioSharing Information Resource), or animal research (see the ARRIVE Guidelines and the STRANGE Framework; for details, see *eLife*'s Journal Policies). Where applicable, authors should refer to any relevant reporting standards materials in this form.

For all that apply, please note **where in the article** the information is provided. Please note that we also collect information about data availability and ethics in the submission form.

Materials:

Newly created materials	Indicate where provided: section/figure legend	N/A
The manuscript includes a dedicated "materials availability statement" providing transparent disclosure about availability of newly created materials including details on how materials can be accessed and describing any restrictions on access.		N/A

Antibodies	Indicate where provided: section/figure legend	N/A
For commercial reagents, provide supplier name, catalogue number and RRID, if available.	Mouse anti-PLAC1 (G-1) (Santa Cruz, Cat# sc-365919; RRID:AB_10846861)<br>• Antibody used in Figure 6D<br>• Antibody information provided in Methods section under the subheading Immunohistochemistry and Image Analysis<br><br>Goat anti-CD31 (R&D Systems, AF3628; RRID:AB_2161028)<br>• Antibody used in Figure 5<br>• Antibody information provided in Methods section under the subheading Immunofluorescence and Image Analysis	

DNA and RNA sequences	Indicate where provided: section/figure legend	N/A
Short novel DNA or RNA including primers, probes: Sequences should be included or deposited in a public repository.		N/A

Cell materials	Indicate where provided: section/figure legend	N/A
Cell lines: Provide species information, strain. Provide accession number in repository OR supplier name, catalog number, clone number, OR RRID.		N/A
Primary cultures: Provide species, strain, sex of origin, genetic modification status.		N/A

Experimental animals	Indicate where provided: section/figure legend	N/A
Laboratory animals or Model organisms: Provide species, strain, sex, age, genetic modification status. Provide accession number in repository OR supplier name, catalog number, clone number, OR RRID.	*Zp3Cre* mice (C57BL/6-Tg 93knw/J; Jackson Labs line 003651; RRID:IMSR_JAX:003651)<br><br>*Eed* floxed mice (*Eed*$^{fl/fl}$) (B6; 129S1-*Eed*tm1Sho/J; Jackson Labs line 0022727; no RRID)<br><br>• Mouse model described in Figure Legend 1A<br>• Mouse model information provided in Methods section under the subheading "Mouse strains, animal care and ethics"	
Animal observed in or captured from the field: Provide species, sex, and age where possible.		N/A

Plants and microbes	Indicate where provided: section/figure legend	N/A
Plants: provide species and strain, ecotype and cultivar where relevant, unique accession number if available, and source (including location for collected wild specimens).		N/A
Microbes: provide species and strain, unique accession number if available, and source.		N/A

Human research participants	Indicate where provided: section/figure legend) or state if these demographics were not collected	N/A
If collected and within the bounds of privacy constraints report on age, sex, gender and ethnicity for all study participants.		N/A

Design:

Study protocol	Indicate where provided: section/figure legend	N/A
If the study protocol has been pre-registered, provide DOI. For clinical trials, provide the trial registration number OR cite DOI.		N/A

Laboratory protocol	Indicate where provided: section/figure legend	N/A
Provide DOI OR other citation details if detailed step-by-step protocols are available.		N/A

Experimental study design (statistics details) *		
**For in vivo studies: State whether and how the following have been done**	**Indicate where provided: section/figure legend. If it could have been done, but was not, write "not done"**	**N/A**
Sample size determination	Power calculations were not performed as the predicted variation between samples was unknown. However, large sample sizes sufficient to facilitate robust statistical analyses were collected. Sample sizes are provided in figure legends e.g. Figure legend of Fig. 2B. N=11-117/genotype and G-H 30-50/genotype. For Fig 6 and an N=4-5/genotype was used for RNAseq and RRBS. For Fig 7 an N=8-12 was used for the metobolomic analyses of fetal samples and all mothers available were analysed. These are a relatively high biological replicates for all of these experiments.	
Randomisation		N/A

Blinding	Where possible, investigators were blinded for sample analyses. Eg for investigators were blinded genotypes throughout quantitative scoring of placental samples. Other samples such as fetal weights were collected from timed mated females of known genotypes as necessary. All data was analysed without bias and without exclusion of datapoints.	
Inclusion/exclusion criteria		N/A

**Sample definition and in-laboratory replication**	**Indicate where provided: section/figure legend**	**N/A**
State number of times the experiment was replicated in the laboratory.	All experimental data is provided as biological replicates. Each replicate represents the phenotypic outcome of an individual animal and therefore an individual replicate. Biological replicates are denoted as the N for each genotype in each experiment and are detailed in the figure legends.	
Define whether data describe technical or biological replicates.	Biological replicates are defined as individual embryos, fetuses or pups (ie individual animals). All experiments include animals from separate litters/pregnancies and with at least 3 different mothers used, though in some cases more than one offspring animal was used from a single litter/pregnancy (eg N= 10 could include 3 fetuses from one litter, 3 from another and 4 from a third litter). Biological replicates used are described in each figure legend. All offspring samples were collected from at least three different females / genotype. eg. Figure legend of Fig. 4A "Images are representative of an individual placenta typical of each genotype, with 10 biological replicates analysed/genotype."	

**Ethics**	**Indicate where provided: section/submission form**	**N/A**
Studies involving human participants: State details of authority granting ethics approval (IRB or equivalent committee(s), provide reference number for approval.		N/A

Studies involving experimental animals: State details of authority granting ethics approval (IRB or equivalent committee(s), provide reference number for approval.	Monash University and Hudson Institute MMCAF Animal Ethics Committee (AEC) guidelines, under approval number MMCB/2018/16<br>• Methods section under "Mouse strains, animal care and ethics and in Declarations	
Studies involving specimen and field samples: State if relevant permits obtained, provide details of authority approving study; if none were required, explain why.		N/A

**Dual Use Research of Concern (DURC)**	**Indicate where provided: section/submission form**	**N/A**
If study is subject to dual use research of concern regulations, state the authority granting approval and reference number for the regulatory approval.		N/A

Analysis:

**Attrition**	**Indicate where provided: section/figure legend**	**N/A**
Describe whether exclusion criteria were pre-established. Report if sample or data points were omitted from analysis. If yes, report if this was due to attrition or intentional exclusion and provide justification.	No exclusion criteria were pre-established as we did not expect to exclude any offspring from the study. All samples collected for successful experiments were included in the analyses.	

**Statistics**	**Indicate where provided: section/figure legend**	**N/A**
Describe statistical tests used and justify choice of tests.	Statistical analyses were performed using GraphPad Prism according to the statistical triage provided in the package. In brief, for parametric tests one way ANOVA with multiple comparison testing was used, with Student's *t* tests for pairwise comparisons included. If indicated in GraphPad Prism, non-parametric were applied. Tests used are indicated in figure legends.	

**Data availability**	**Indicate where provided: section/submission form**	**N/A**
For newly created and reused datasets, the manuscript includes a data availability statement that provides details for access (or notes restrictions on access).	All datasets are described below and in the Declarations section of the manuscript under the subheading "Availability of data and materials": All RNA sequencing data have been deposited to the Gene Expression Omnibus (GEO) and are available to reviewers via accession number GSE210398 and an access token as described below.<br><br>To review GEO accession GSE210398: Go to https://www.ncbi.nlm.nih.gov/geo/query/acc.cgi?acc=GSE210398 Enter token grqbguasddiffqf into the box.<br><br>Upon acceptance of the manuscript, all RNAseq data will be immediately released for public access. This will include all source data files containing raw data counts and associated metadata.	
When newly created datasets are publicly available, provide accession number in repository OR DOI and licensing details where available.	E17.5 mouse placenta RNA-seq accession number GSE210398.<br>• Figure 6<br>• Accession number in declarations section	
If reused data is publicly available provide accession number in repository OR DOI, OR URL, OR citation.		N/A

**Code availability**	**Indicate where provided: section/figure legend**	**N/A**
For any computer code/software/mathematical algorithms essential for replicating the main findings of the study, whether newly generated or re-used, the manuscript includes a data availability statement that provides details for access or notes restrictions.		N/A
Where newly generated code is publicly available, provide accession number in repository, OR DOI OR URL and licensing details where available. State any restrictions on code availability or accessibility.		N/A

If reused code is publicly available provide accession number in repository OR DOI OR URL, OR citation.		N/A

Reporting:

The MDAR framework recommends adoption of discipline-specific guidelines, established and endorsed through community initiatives.

**Adherence to community standards**	**Indicate where provided: section/figure legend**	**N/A**
State if relevant guidelines (e.g., ICMJE, MIBBI, ARRIVE, STRANGE) have been followed, and whether a checklist (e.g., CONSORT, PRISMA, ARRIVE) is provided with the manuscript.		N/A

---

* We provide the following guidance regarding transparent reporting and statistics; we also refer authors to Ten common statistical mistakes to watch out for when writing or reviewing a manuscript.

Sample-size estimation

- You should state whether an appropriate sample size was computed when the study was being designed
- You should state the statistical method of sample size computation and any required assumptions
- If no explicit power analysis was used, you should describe how you decided what sample (replicate) size (number) to use

Replicates

- You should report how often each experiment was performed
- You should include a definition of biological versus technical replication
- The data obtained should be provided and sufficient information should be provided to indicate the number of independent biological and/or technical replicates
- If you encountered any outliers, you should describe how these were handled
- Criteria for exclusion/inclusion of data should be clearly stated
- High-throughput sequence data should be uploaded before submission, with a private link for reviewers provided (these are available from both GEO and ArrayExpress)

Statistical reporting

- Statistical analysis methods should be described and justified
- Raw data should be presented in figures whenever informative to do so (typically when N per group is less than 10)
- For each experiment, you should identify the statistical tests used, exact values of N, definitions of center, methods of multiple test correction, and dispersion and precision measures (e.g., mean, median, SD, SEM, confidence intervals; and, for the major substantive results, a measure of effect size (e.g., Pearson's r, Cohen's d)

- Report exact p-values wherever possible alongside the summary statistics and 95% confidence intervals. These should be reported for all key questions and not only when the p-value is less than 0.05.

Group allocation

- Indicate how samples were allocated into experimental groups (in the case of clinical studies, please specify allocation to treatment method); if randomization was used, please also state if restricted randomization was applied
- Indicate if masking was used during group allocation, data collection and/or data analysis
